# Landscape Evolution and It’s Impact of Ecosystem Service Value of the Wuhan City, China

**DOI:** 10.3390/ijerph182413015

**Published:** 2021-12-09

**Authors:** Ru Chen, Chunbo Huang

**Affiliations:** 1 School of Arts and Communication, China University of Geosciences, Wuhan 430078, China; chenru@cug.edu.cn; 2 Research Center of Spatial Planning and Human-Environmental System Simulation, School of Geography and Information Engineering, China University of Geosciences, Wuhan 430078, China

**Keywords:** ecological elements, human activities, urban expansion, land use, waterscape, landscape planning

## Abstract

Rapid urbanization and industrialization and enhanced ecological protection measures have greatly influenced landscape change, which has exacerbated regional landscape competition and conflicts and indirectly affected the supply of ecosystem services. Clarifying the relationship between ecosystem service change and landscape change is useful for understanding the impact of ecosystem conversion on socio-economic development and providing a knowledge base for relevant policy decisions. In this study, we used remote sensing technology to process Landsat TM/ETM+/OLI imageries, combined with transformation analysis and kernel density analysis to study the spatial and temporal characteristics of land use change in Wuhan City from 1980 to 2020. We also estimated the ESV in the region using the improved unit area value equivalent method to reveal the trends of ESV changes in Wuhan. The results showed that land use changes in Wuhan during 1980–2020 occurred mainly in terms of decreases in farmland, forestland, and bare land, as well as increases in built-up land and water bodies. The built-up land was mainly concentrated in the main urban areas, but its area in each suburban area has increased in recent years. In contrast, farmland was mainly distributed in suburban areas, and its area has been decreasing in recent years due to the impact of urban expansion. However, the reduction is compensated for by the reclamation of ecological land such as grassland and forestland, which has aggravated the loss of ecosystem service values in the study area. In addition, human activities such as urban expansion have increased the demand for water resources, while also leading to ecological problems such as water scarcity and water quality degradation, which have caused serious losses to key ecosystem services in Wuhan city. Therefore, in order to alleviate the competition and conflicts in the landscape and mitigate the loss of ecosystem service values in this area, we have proposed some constructive suggestions for future urban planning and water quality improvements in Wuhan. The focus of these suggestions is on controlling the expansion of built-up land, as well as the conservation of ecological land and resource protection. Meanwhile, our findings can also provide reference information for land resource planning and ecological monitoring, and help researchers to understand the contribution of ecosystem service functions in relation to socio-economic development.

## 1. Introduction

Landscape change is the basis of resource, environmental, and ecological research and scientific management, and it directly reflects the response of regional ecosystem structures and processes to environmental change [1,2]. It not only objectively records the spatial patterns of the earth’s surface features that are altered by human activities, but also reproduces the spatial and temporal dynamics of the earth’s surface landscape [3]. Since the reform and opening up of China, rapid urbanization and industrialization, as well as enhanced ecological protection measures, have led to tremendous pressure and challenges in relation to landscapes in China, which have in turn greatly influenced landscape changes [4,5]. For example, Li et al. [6] used geographically weighted regression (GWR) to identify the spatial non-stationary relationship between urbanization and landscape patterns in Beijing City, China, demonstrating that urbanization would result in the expansion of built-up land and thus make the landscape more fragmented and irregular. Li et al. [7] used recently developed built-up data and land use data to describe the impact of human settlement expansion on landscape fragmentation and its spatio-temporal heterogeneity in China between 1975 and 2014, finding that the mean built-up area ratio increased by three times over the 40 years in China, which resulted in a significant change in the landscape fragmentation pattern. However, landscape change often generates a variety of environmental problems that aggravate regional landscape competition and conflicts, thus indirectly affecting the supply of ecosystem services [8,9]. It can also have an impact on the goods and services provided by ecosystems by altering their type and intensity [10]. Therefore, it is important to quantitatively describe the complex spatial and temporal processes of landscape changes and to further reveal the relationships between ecosystem service changes and different landscapes. This is beneficial for understanding their ecological and economic relationships, which can provide reference information for land resource planning and eco-environmental monitoring [2,11].

Landscape change driven by urbanization is a major contributor to changes in the spatio-temporal dynamics of ecosystems and is a major factor driving ecosystem service losses [12,13]. The ecosystem service value (ESV) is the value of goods and services provided by ecosystems for human well-being, and is the main indicator for assessing ecosystem change. It quantifies the direct or indirect human benefits from ecosystem functions [14,15]. Currently, some studies (e.g., Cai et al. [16] and Hu et al. [17]) have demonstrated the relationship between urbanization and ESV, suggesting that urbanization indirectly leads to ESV changes by affecting the structure and function of natural ecosystems. Peng et al. [18] also found that urbanization can affect the flow of materials, energy, and information by changing landscape patterns, which in turn changes the services provided by regional ecosystems to humans. However, most studies have only used a single or several ecosystem services to assess the response of ESV to urbanization. The intrinsic linkages among various ecosystem services remain unclear. Therefore, an integrated study of ESV changes in various ecosystems is necessary to help understand the contribution of ecosystem service functions to socioeconomic development and provide a knowledge base for relevant policy decisions.

Costanza et al. [19] first proposed an ecosystem assessment method and estimated 17 ecosystems provided by 16 land resources [16,20]. This caused ESV to be widely studied globally, and it has become a research hotspot for environmental impact assessments [21,22]. However, this method has high requirements in terms of data collection and processing, and the calculation is relatively complicated and is not generalized [23]. Therefore, Xie et al. [24] conducted a questionnaire survey of domestic ecologists and revised the coefficient table based on that of Costanza et al. [19]. Compared with other methods, this method is considered more practical and has the advantages of low data requirements, high comparability of results, and the ability for comprehensive evaluation, and it has been widely used for ESV assessments in China [15,25,26]. Therefore, in this study we selected 11 ecosystem services to study the trends of ESV changes based on the classification system of Xie et al. [24].

Wuhan city is the largest city in the central part of China, with a unique geographical location and a good economic foundation [10,27]. It is currently recognized as the center of finance, education, and transportation in central China, and has been identified as a core region of the Central Rising Strategy and China’s Yangtze River Economic Belt Development Strategy [20]. This has driven the evolution of Wuhan’s spatial pattern in the landscape, leading to a rapid urbanization period with large-scale population migration [28,29]. However, this evolution has exposed the landscape to the pressures and challenges of the continuous expansion of built-up land and the accelerated degradation of ecological land, and has brought about many eco-environmental problems, such as lake reclamation, water pollution, and farmland erosion [5,25]. These human activities have disrupted the ecological balance of the lake and reduced its self-regulating capacity, thus leading to frequent urban flooding and even inducing catastrophic weather in local areas [21,30]. In addition, the expansion of built-up land at the cost of ecological land has led to a huge loss of high-quality farmland and a drastic degradation of ecosystem services in Wuhan [21,31]. Therefore, it is urgent to study the dynamic changes and transfer probabilities of ecological land in order to alleviate the landscape conflicts, improve the ecological environment, as well as to maintain sustainable development [5,32], so as to provide a scientific basis for the optimization of ecological land in Wuhan and the formulation of urban planning.

To understand the trends of landscape changes in Wuhan during 1980–2020, we studied the spatial and temporal characteristics of the landscape, using four indicators (net total change area, annual change area, annual change rate, and dynamic degree) and kernel density analysis. The conversion matrix was used to determine the conversion direction of various land use types. In addition, we estimated the ESV of the region at different periods using an improved unit area value equivalent method to reveal the trends of ESV changes in Wuhan city. The specific objectives of this paper are as follows: (1) to reveal the landscape pattern evolution characteristics of the landscape in Wuhan under the influence of human activities such as urban expansion; (2) to analyze the trends in ESV changes due to landscape changes in Wuhan city during 1980–2020; (3) to provide scientific suggestions and a theoretical basis for urban expansion and watershed improvements in Wuhan City by combining landscape changes with ESV trends.

## 2. Study Area

**Figure 1:**
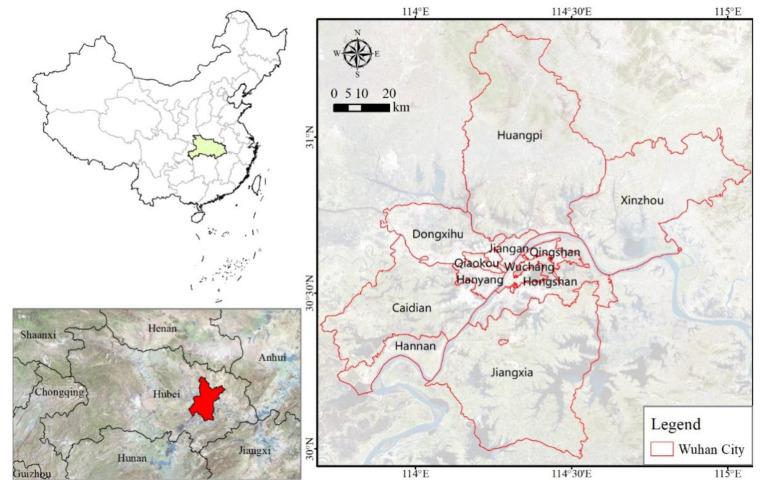
The geographical location of Wuhan city, China.

Wuhan City is located in the eastern part of the Jianghan Plain, at the confluence of the Yangtze River and the Han River (Figure 1). It lies between 29°58′–31°22′ N and 113°41′–115°05′ E. It borders Huanggang City and Ezhou City to the east, Jingzhou City to the south, Xiantao City to the west, and Xiaogan City to the north. Wuhan is the capital of Hubei Province and is an important industrial base, a science and education base, and a comprehensive transportation hub in China. It has a total area of 8494 km^2^ and includes 7 main urban areas (Jiangan District, Jianghan District, Qiaokou District, Hanyang District, Wuchang District, Qingshan District, and Hongshan District) and 6 suburban areas (Caidian District, Jiangxia District, Dongxihu District, Hannan District, Huangpi District, and Xinzhou District). The topography is predominantly plain, with flat terrain and abundant water resources. Water accounts for about a quarter of the entire city, setting it apart from other cities in China [4]. The region is typical of a north subtropical monsoon climate with abundant rainfall, sufficient heat, and four distinct seasons. Its average annual temperature is 15.8 °C–17.5 °C, and the annual precipitation is 1150–1450 mm.

Wuhan has been the most economically active region in central China since the country began its economic reform and opening up to the outside world in the late 1970s [10]. Thus, its urban area is expanding and the population is increasing year by year, which has a strong influence on the socio-economic development of the surrounding areas. In addition, the implementation of a series of planning measures has exacerbated the imbalance of regional development within Wuhan, thereby affecting the city’s landscape and urban expansion in the coming years [29]. Meanwhile, high-intensity land development has also caused various ecological problems, such as endangering biodiversity, reducing water quality, disrupting soil cycles, and degrading local wetland ecosystems [33]. These changes inevitably alter the ecosystem service function of the land, thereby negatively impacting the goods and services provided by the ecosystem [10]. Therefore, it is necessary to study thoroughly the spatial and temporal evolution and intrinsic linkages of landscape changes and ecosystem services in Wuhan to find a reasonable trade-off between economic development and ecological conservation.

## 3. Study Data and Methods

### 3.1. Data Sources

Landscape maps of Wuhan city in 1980, 1990, 2000, 2010, and 2020 were derived from the 30-m-resolution Landsat TM/ETM+/OLI imageries, which were obtained from the Data Cloud of CAS (http://www.csdb.cn/, accessed on 1 May 2021). Six land-use types (Table 1) were identified: farmland, forestland, grassland, water, built-up land, and bare land.

### 3.2. Statistical Analysis

#### 3.2.1. Indicators of Landscape Change

In order to analyze the landscape change characteristics, four indicators (the net total area of change, the annual area of change, the annual rate of change, and the dynamic degree) were used for each land use change type. We divided the period 1980–2020 into four phases with an interval of 10 years, i.e., 1980–1990, 1990–2000, 2000–2010, and 2010–2020. The net total area of change is the total area change of each land use type from 1980 to 2020. The annual area of change is the area change of land use types in the four phases.

We used the following equation to calculate the annual rate of change (*K_i_*) of the land use type of *i*.

$$K_{i}=\frac{1}{t}*\left\{\sum_{j}^{n}\left(\frac{∆S_{i,j}}{S_{t}}\right)\right\}*100\%$$

where *S_t_* is the area of *i* at the start of monitoring, Δ*S_i,j_* is the total net area from the other changed land use types *j* to *i*, and *t* is the time period. *K_i_* reflects the annual change rate of *i* within the study area during *t*.

Dynamic degree refers to the change in the land use type in the study area over a certain time interval, which could truly reflect the drastic changes of land use types in the regional land use/cover. In this paper, the following equation was used to calculate the dynamic degree (*S_i_*) of the land use type of *i*.

$$S_{i}=\frac{1}{t}*\left\{\sum_{j}^{n}\left(\frac{\left|∆S_{i,j}\right|}{S_{a}}\right)\right\}*100\%$$

where *S_a_* is the total study area and |Δ*S_i,j_*| is the absolute value of the total area from the other changed land use types *j* to *i*. *S_i_* reflects the change intensity of *i* within the study area during *t*.

#### 3.2.2. Kernel Density Analysis

Kernel density analysis is a process of interpolating through discrete points. The points that fall into the search area have the same weight. Kernel density analysis can generate a continuous surface to reflect the point aggregation of the entire region, which can reflect the spatial distribution characteristics. In this paper, we used the ArcGIS software to convert the landscape change pixels into vector data, and calculated the kernel density of urban expansion and increased water through the “Kernel Density” tool. According to the landscape characteristics, we determined through testing that the optimal search radius for kernel density analysis in this study should be set at 2 km.

#### 3.2.3. Transformation Analysis for the Land Use Type

The matrix tool in ERDAS IMAGINE allows one to check any change in land cover classes between certain times by applying a post-classification change-detection algorithm to the classified images [34]. The obtained transition matrix is a table containing a systematic array of land cover categories from the initial phase to the final phase [35]. To identify the main conversion directions and highlight the dominant dynamic events in the landscape changes, we used the ERDAS IMAGINE matrix tool (Version 9.2, ERDAS, Redlands, CA, USA) to generate transfer matrices in different periods.

$$A_{ij}=\left[\begin{array}{cc} \begin{array}{cc} A_{11} & A_{12}\\ A_{21} & A_{22} \end{array} & \begin{array}{cc} \cdots & A_{1n}\\ \cdots & A_{2n} \end{array}\\ \begin{array}{cc} \vdots & \vdots \\ A_{n1} & A_{n2} \end{array} & \begin{array}{cc} \ddots & \vdots \\ \ldots & A_{nn} \end{array} \end{array}\right]$$

*A_ij_* is the area percentage (%) of the land use type converted from *i* to *j* during the period, which could reveal the distinct transformation phases. *n* is the total number of the land use types and this equaled six in this study.

### 3.3. Assessing Ecosystem Service Value

Ecosystem services are derived from the flow of goods, energy, and information from ecosystems. Assessing the ESV requires breaking down complex structures and processes into a limited number of functions. These functions are required to produce direct and indirect benefits that represent the human benefit from the ecosystem, including resource provisioning, environmental regulation, cultural recreation, and production support [24]. To assess the impacts of urbanization and economic development on local ecosystems, in this study we assessed 11 ecosystem services, including 2 direct ecosystem services and 9 indirect ecosystem services. Based on the ecosystem service value equivalence coefficients proposed by Costanza et al. [19] and the “Ecosystem service value equivalence scale for terrestrial ecosystems in China” by Xie et al. [24], we revised the ecosystem service value equivalence for Wuhan city. The per-unit standard value of ecosystem services is the economic value of the grain produced by an average hectare of farmland [24]. To quantify the contribution of various ecosystems to ecosystem services, we considered the net profit per unit of food production in farmland ecosystems as the value per unit of standard ecosystem services. Based on this and various ecosystem service value coefficients, we estimated the total value of ecosystem services. The formula used for this calculation was as follows:

$$D=\sum_{i=1}^{n}S_{i}*F_{i}$$

where *D* is the value of the ecosystem service value equivalent factor ($/ha). *S_i_* is the percentage of the crop area of *i* (%). *F_i_* is the average net profit per unit area for crop of *i* ($/ha); and the range of *i* is [1, n].

According to the national statistical data for crops in 2010 selected by Xie et al. [24], we calculated the D value, and the result was 484.64 USD/ha. Furthermore, we multiplied the D value with the equivalence coefficient of ESV per unit area to obtain the ecosystem service equivalent value per unit area (Table 2). The ESV for each land use type was obtained by multiplying the area of each land use with the corresponding value equivalent. The total ESV was obtained by summing up the ESVs of 11 ecosystem services for each land use type.

## 4. Results

### 4.1. The Landscape Change Assessment

Based on the area of six land use types in Wuhan (Table 3), it can be concluded that farmland, forestland, and water were the main landscapes during 1980–2010, accounting for about 90% of the total area. Furthermore, during 2010–2020, farmland, water, and built-up land were the main landscapes, accounting for about 88% of the total area. Between 1980 and 2020, the areas of farmland, forestland and bare land decreased year by year, from 5714.93 km^2^, 817.32 km^2^, and 146.48 km^2^ to 4526.66 km^2^, 773.85 km^2^, and 60.95 km^2^, respectively. Meanwhile, the area of built-up land was growing dramatically, from 495.83 km^2^ in 1980 to 1319.82 km^2^ in 2020. In addition, the change in the water area showed an overall upward trend during the past 40 years, and its area was the largest in 2010, at 1845.49 km^2^. Grassland area declined yearly during 1980–2000 and increased yearly during 2000–2020, with no significant change overall.

Through the changes in the areas of the six land use types in each phase from 1980 to 2020 (Table 4), we found that the land use changes in Wuhan at different development stages showed different patterns. During 1980–2010, the land use change was dominated by the conversion between built-up land, watershed, and other land use types. The land use change during 2010–2020 mainly involved the transfer of other land use types to built-up land. Specifically, farmland area decreased the most during 2000–2010, with a decrease of 479.84 km^2^. Its area decreased overall by 1188.27 km^2^ during the past 40 years. The area of forestland decreased the most during 1990–2000, by 19.03 km^2^. The next largest decrease was during 2000–2010, by 17.21 km^2^. In terms of grassland, its area decreased between 1980 and 2000, whereas it increased between 2000 and 2020. There was no significant change overall, with a decrease of only 0.66 km^2^. The water area only continued to decline from 2010 to 2020, decreasing by 31.32 km^2^. Its area grew the most from 1980 to 1990, increasing by 351.35 km^2^. Overall, its area has grown by 494.06 km^2^ over the past 40 years. In addition, the area of built-up land increased by 823.99 km^2^ between 1980 and 2020, with the largest increase of 394.60 km^2^ occurring during 2000–2010. In contrast, the area of bare land decreased by 85.53 km^2^ over the past 40 years, with the largest decrease of 61.74 km^2^ occurring during 1980–1990.

From 1980 to 2020, the most significant land use change in Wuhan was in built-up land, with an annual change rate of 4.15% (Table 5). This was followed by bare land, with an annual rate of change of −1.46%. The annual rates of change in farmland and water bodies were similar, but they were in opposite directions, at −0.52% and 0.94%, respectively. In addition, there was a slight decrease in forestland and grassland. For the different development phases, farmland had the highest annual rate of change of −0.92% per year during 2000–2010, followed by the 1980–1990 period, with an annual rate of change of −0.58% per year. The highest annual rate of change in forestland area was observed during 1990–2000 and 2000–2010, with −0.23% and −0.22% per year, respectively. The annual rate of change in grassland area was negative during 1980–2000, whereas it was positive during 2000–2020. In addition, the watershed had a negative annual rate of change of −0.17% per year during 2010–2020, whereas it had the highest annual rate of change of 2.66% per year during 1980–1990. The built-up land area had the highest annual change rate of 5.99% per year during 2000–2010. This was followed by 2.53% per year during 2010–2020. The highest annual rate of change in bare land area was −4.21% per year during 1980–1990. Overall, the area of the six land use types changed most dramatically during 2000–2010 and 1980–1990, with dynamic degrees of 1.19% and 0.95%, respectively.

### 4.2. Spatial Analysis of the Landscape Change

**Figure 2:**
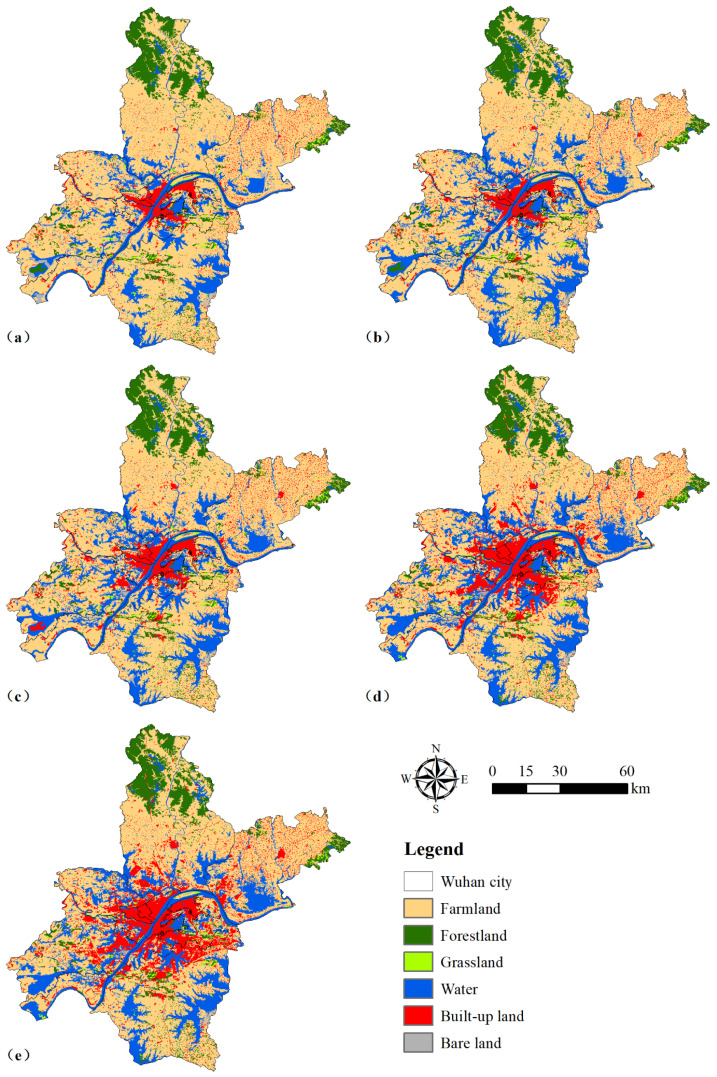
Spatial distribution maps of the land use types in 1980 (**a**), 1990 (**b**), 2000 (**c**), 2010 (**d**), and 2020 (**e**) in Wuhan city, China.

According to the spatial distribution maps of the six land use types (Figure 2), we found that the farmland was mainly distributed in the suburban areas, such as Caidian District, Jiangxia District, Dongxihu District, Hannan District, Huangpi District, and Xinzhou District. The forestland was mainly located in the northern part of Huangpi District and the southeastern part of Xinzhou District. Grassland was scattered in the Dongxihu District, Caidian District, Hannan District, Jiangxia District, Hongshan District, and Qingshan District. Moreover, water bodies were mainly in the districts around the main urban areas. There were also many water bodies distributed within the main urban area, such as the Yangtze River and East Lake. The built-up land was concentrated in the main urban areas and scattered in the suburban areas. Although the proportion of bare land area was small, it was sporadically distributed within each district. By comparing the landscape distribution maps for each year, we found that there was a significant expansion of built-up land and water bodies during 1980–2020, whereas the area of farmland decreased significantly.

**Figure 3:**
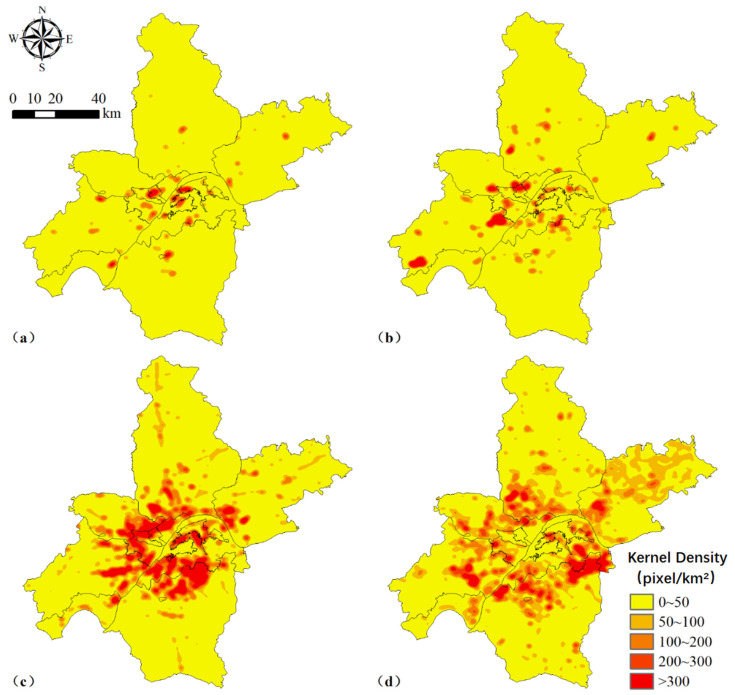
Kernel density of urban expansion during 1980–1990 (**a**), 1990–2000 (**b**), 2000–2010 (**c**), and 2010–2020 (**d**) in Wuhan city, China. Each pixel represents 30 m × 30 m.

Based on the kernel density analysis map of urban expansion, urban expansion activities during 1980–1990 were mainly concentrated in Wuchang District, Qingshan District, and Qiaokou District (Figure 3a). There were also small amounts of urban expansion activities within other districts. Between 1999 and 2000, urban expansion activities were sporadically distributed within the districts, mainly including Qiaokou District, Jiangan District, and Caidian District (Figure 3b). Comparing the kernel density analysis maps for the four periods, there was a significant increase in the aggregation of built-up land within the main urban areas between 2000 and 2010 (Figure 3c). It was mainly distributed in Wuchang District, Hongshan District, Hanyang District, Qiaokou District, Jiangan District, Jiangxia District, Caidian District, Dongxihu District, Huangpi District, and Xinzhou District. In contrast, the aggregation of built-up land within each suburban area increased significantly during 2010–2020, especially in Jiangxia District, Caidian District, Dongxihu District, and Huangpi District (Figure 3d). Other areas (e.g., Xinzhou District and Hannan District) also experienced some degree of urban expansion during the study period.

**Figure 4:**
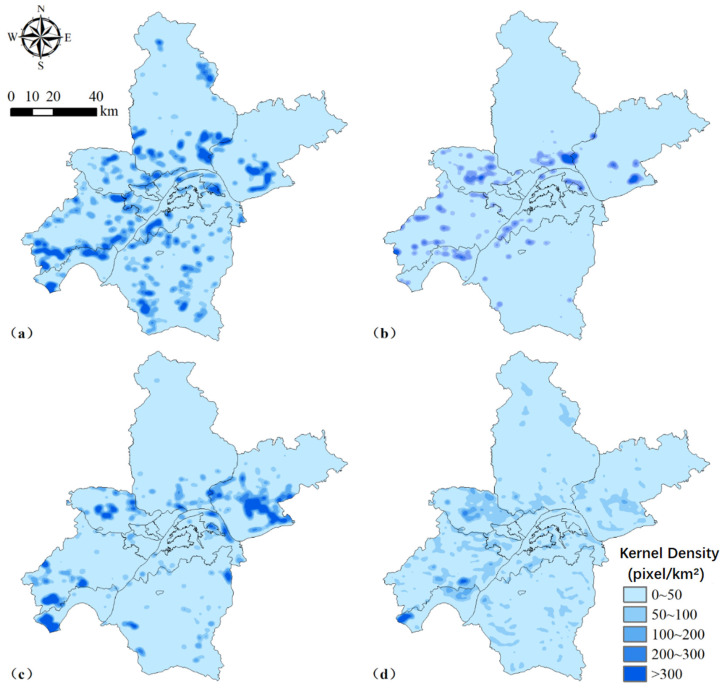
Kernel density of increased water during 1980–1990 (**a**), 1990–2000 (**b**), 2000–2010 (**c**), and 2010–2020 (**d**) in Wuhan city, China. Each pixel represents 30 m × 30 m.

Similarly, we concluded from the kernel density analysis map that the water increased the most during 1980–1990 (Figure 4a). The increase in water was observed in all areas except the northern part of Huangpi District and the northeastern part of Xinzhou District. During 1990–2000, the increase in water was mainly distributed in Dongxihu District, Caidian District, Hannan District, Jiangxia District, and Qingshan District (Figure 4b). The southern part of Huangpi District and Xinzhou District also experienced a portion of this water body expansion. In addition, the areas of increased water during 2000–2010 were mainly concentrated in Dongxihu District, Caidian District, Hannan District, Jiangxia District, Xinzhou District, Qingshan District, and the southern part of Huangpi District (Figure 4c). During 2010–2020, the water bodies in each district were growing (Figure 4d). However, the trend of increased water was more moderate in this period compared to several other periods.

### 4.3. Transformation Analysis of Land Use Types

**Figure 5:**
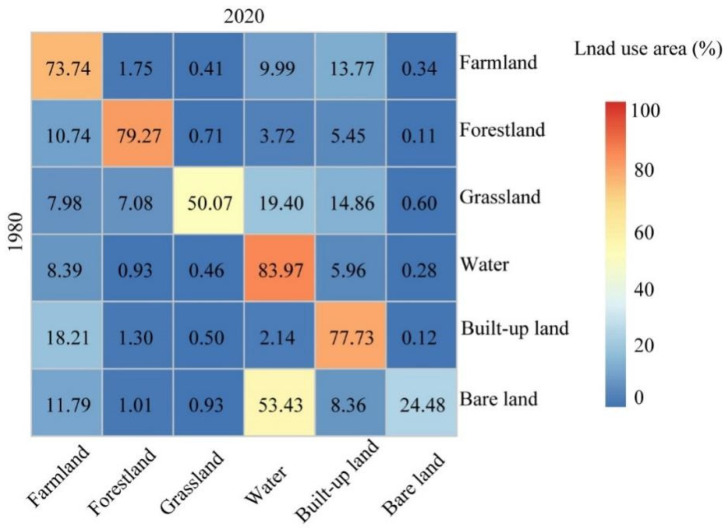
Landscape transformation (land use area %) of Wuhan city between 1980 and 2020. These data were derived from spatial distribution maps in 1980 and 2020. In each row, the numbers indicate the area percentage of a land use type (row header) converted to another type (column header) during the period, and the sum is 100. In each column, the data document a land use type (column header) that was converted from other types (row headers) during the period.

The landscape transformation revealed that the transformation of the landscape in Wuhan mainly occurred between construction land, water bodies, and farmland and bare land (Figure 5). During 1980–2020, the increase in built-up land mainly originated from grassland, farmland and bare land, with transfer rates of 14.86%, 13.77%, and 8.36%, respectively. The extended water bodies mainly came from bare land, grassland, and farmland, with transfer rates of 53.43%, 19.40%, and 9.99%, respectively. In addition, the transfer rates of built-up land, bare land, and forestland to farmland were 18.21%, 11.79%, and 10.74%, respectively, whereas the transfer-out rates from grassland and farmland to forestland were 7.08% and 1.75%, respectively. Most of the farmland and grassland was converted to built-up land and water, whereas forestland was mostly converted to farmland. In addition, 83.97%, 79.27%, and 77.73% of the water bodies, forestland, and built-up land remained unchanged during 1980–2020. Overall, the transfer-in rates of built-up land and water bodies were higher, whereas the transfer-out rates of grassland and bare land were relatively high.

### 4.4. Ecosystem Service Value Changes in Wuhan City

The total ESV in the study area was increased during 1980–2010 and then decreased during 2010–2020 (Table 6). Its total ESV was the highest in 2010 with a value of USD 1.31 × 10^10^. In terms of ecosystem service type, the ESV of farmland ecosystem showed a decreasing trend from USD 1.08 × 10^9^ to USD 8.57 × 10^8^ during 1980–2020. In contrast, the ESV of forestland ecosystem decreased during 1980–2010 and increased during 2010–2020. Its ESV was the highest in 1980 with a value of USD 9.89 × 10^8^. The wetland ecosystem had the highest ESV of all ecosystems, accounting for about 80% of the total ESV. It showed a continuous increasing trend during 1980–2020, from USD 8.07 × 10^9^ to USD 1.11 × 10^10^. The ESV of the desert ecosystem was extremely low, accounting for only 0.01% of the total ESV. Its ESV decreased from USD 1.43 × 10^6^ in 1980 to USD 5.93 × 10^5^ in 2020.

According to the ESV changes of different ecosystem services (Table 7), it can be concluded that the ESVs of all ecosystem services in farmland ecosystem decreased during 1980–1990, except for water supply services. The ESVs of hydrological regulation service, food production services, and gas regulation service sdecreased the most, by USD 4.42 × 10^7^, USD 2.21 × 10^7^ and USD 1.80 × 10^7^, respectively. The ESV of water supply services increased by USD 4.27 × 10^7^. The ESVs of 11 ecosystem services in forestland ecosystems were reduced. Climate regulation services, hydrological regulation services, and soil retention services had the largest reductions in ESVs, with reductions of USD 2.85 × 10^6^, USD 2.09 × 10^6^, and USD 1.27 × 10^6^, respectively. Conversely, the ESVs of 11 ecosystem services in wetland ecosystems all increased. The hydrological regulation service and water supply service had the largest increases in ESVs, with increases of USD 1.75 × 10^9^ and USD 1.42 × 10^8^. Desert ecosystem had only six ecosystem services, including gas regulation services, environmental purification services, hydrological regulation services, soil retention services, biodiversity conservation services, and aesthetic landscape services. Their ESVs all decreased during the study period. Environmental purification services showed the greatest reduction in ESV, with a reduction of USD 3.00 × 10^5^. The trends of ESV changes in several other periods were consistent with the 1980–1990 period, differing only in the amount of change. In contrast, the ESVs of each ecosystem service in farmland ecosystems changed the most during 2000–2010. The ESVs of 11 ecosystem services in forestland ecosystems varied the most during 1990–2000, whereas those of wetland and desert ecosystems varied the most during 1980–1990.

In addition, we found significant differences in the overall ESV of each ecosystem service for each period (Table 7). Between 1980 and 1990, the ESVs were increasing in all ecosystem services except for food production services, gas regulation services, and nutrient cycling services. Hydrological regulation services and water supply services had the largest increase in ESV, by USD 1.70 × 10^9^ and USD 1.84 × 10^8^, respectively. The ESV of food production services decreased the most, with a decrease of USD 8.59 × 10^6^. During 1990–2010, only the ESVs of food production services, raw material production services, gas regulation services, climate regulation services, and nutrient cycling services were decreasing, whereas the ESVs of several other ecosystem services were increasing. The ESV of gas regulation services decreased the most between 1990 and 2000, by USD 7.44 × 10^6^. This was followed by the ESV for food production services, which decreased by USD 7.36 × 10^6^. In contrast, the ESV of food production services decreased the most during 2000–2010, by USD 2.77 × 10^7^. The hydrological regulation service and water supply service had the largest increases in ESVs, by USD 4.71 × 10^8^ and USD 1.05 × 10^8^, respectively. In addition, only the ESV of water supply services was increasing during 2010–2020, with an increase of USD 1.66 × 10^7^. The ESVs of other ecosystems were decreasing. Furthermore, hydrological regulation services showed the largest decrease in ESV, with a reduction of USD 1.87 × 10^8^.

## 5. Discussion

### 5.1. Temporal Trends of the Landscape Change

During 1980–2020, there were obvious changes in land use changes in Wuhan (Table 3). The characteristics of land use changes differed significantly in different development phases (Table 4). During 1980–2010, the main characteristic was the interconversion between farmland, forestland, and bare land with water bodies and built-up land. However, the conversion of other land use types to built-up land was the main characteristic during 2010–2020. Similar results were found by Min et al. [36]. This was mainly attributed to the disturbance of human activities and the rapid economic development in Wuhan [37]. Wuhan is a metropolis, known for its abundant water resources, so the local government has emphasized the importance of protecting the watershed and maintaining its unique characteristics [4], which is the main reason for the increasing area of water bodies during the study period. However, human activities such as lake-filling projects in pursuit of industrial development during 2010–2020 led to a significant shrinkage of the water area in Wuhan, which weakened the water storage capacity of the lake and posed a certain threat to the local wetland ecosystem [33]. Furthermore, in order to pursue economic benefits, China’s early urbanization policies largely neglected ecological protection and the sustainable utilization of natural resources [21,38]. The Wuhan Urban Master Plan states that the urbanization level of Wuhan should reach 84% by 2020, which promotes the transfer of other land use types to built-up land and intensifies the competition between economic benefits and ecological protection [4,17]. In addition, the large-scale migration of China’s rural population to urban areas has led to the expansion of built-up land. However, the area of rural built-up land has not been reduced, leading to an imbalance in landscape structure and ecological environment deterioration [4].

To protect farmland and improve the environment, the Chinese government has implemented a series of land policies and carried out six National Forestry Ecological Construction Projects over the past 40 years, mainly including the requisition–compensation balance of farmland (RCBF) policy and reforestation policy [39]. However, these policies require developers to reclaim farmland area occupied by built-up land elsewhere [30]. Therefore, compensation for the loss of farmland due to built-up land expansion can lead to additional ecological land losses (e.g., of forestland, bare land, and grassland), which in turn hinders economic development [12]. This result was also supported by the annual rate of change for various land use types (Table 5) and the landscape transformation of Wuhan city between 1980 and 2020 (Figure 2). It confirmed that urban sprawl and rapid economic growth have greatly increased the demand for natural resources [40]. This was similar to the findings of Hu et al. [17], who found that urban expansion must come at the cost of the loss of natural or semi-natural landscapes, resulting in serious impacts on the ecological environment. On the one hand, continued urbanization increases the impervious surface area and reduces vegetation cover, such as grassland and woodland, thus increasing the risk of urban flooding [20]. On the other hand, Wuhan city has intensified the wasting of resources and environmental pollution to improve its economic power and people’s living standards since 2006 [27]. Therefore, although urbanization promotes socioeconomic development and improves the quality of life, the transition from semi-natural and natural landscapes to impermeable surfaces can also cause significant environmental and ecological problems [15]. Therefore, it is necessary to develop reasonable landscape planning to improve regional ecological quality and ecosystem services by changing landscape patterns and structures [41]. The focus should be on controlling the expansion of built-up land, as well as the reduction of ecological land area such as that of grasslands and forestlands.

### 5.2. Spatial Characteristics and ESV Dynamics among These Land Use Types

Since 1980, rapid socio-economic development and urbanization have led to large changes in the spatial and temporal patterns of the landscape in Wuhan (Figure 2). Farmland was mainly distributed in suburban areas, whereas water landscapes such as rivers and lakes were interspersed in the periphery of the main urban area. In contrast, the distribution of built-up land was more concentrated and mainly located in the central urban area of Wuhan, and forestland was mainly clustered in the northern part of the study area, which was consistent with the findings of Li et al. [42]. Moreover, there was a significant trend of land expansion in the central city of Wuhan during 1980–2020 (Figure 3). The area of water bodies distributed in the suburban areas was also increasing, but that in the main urban areas was continuously decreasing due to the expansion of built-up land (Figure 4). Similar results were found by Wang et al. [31] and Peng et al. [43]. The former demonstrated that the decrease in farmland area was spatially consistent with the increase in built-up land, indicating that the urban expansion was encroaching on farmland. The latter emphasized that urbanization has posed a serious threat to the survival conditions of urban wetlands. There were two main reasons for this spatial evolution pattern in Wuhan City. One reason is that the flat topography of Wuhan provides a wide space for landscape expansion, which has led to a rapid increase in built-up land and industrial land, as well as a dramatic decrease in lakes and farmland around the main urban area [44]. Another reason is because Wuhan city, as a national lake reserve and a national ecological representative area, must protect the watershed and the unique ecosystem to maintain national ecological security [4,27]. However, the rapid development of urbanization has also brought about a series of environmental and resource problems that constrain the sustainable development of Wuhan’s economy, society, and ecological environment [40].

Rapid population growth and urban expansion have also led to the loss or destruction of key ecosystem services [37]. The study showed that the total ESV in Wuhan gradually increased during 1980–2010 but decreased during 2010–2020 (Table 6). This was mainly attributed to the urban expansion of Wuhan city in recent years and the loss of water bodies during 2010–2020, which was supported by Wang et al. [20]. On the one hand, human activities such as lake filling break the ecological balance of lakes and reduce their self-regulation and water storage capacity [21]. On the other hand, built-up land in densely populated areas expands under the influence of urban economic development, which eventually leads to the loss of ESV [25]. Furthermore, the expansion of built-up land has reduced the farmland area, thus reducing the supply capacity of ecosystem services and regional biodiversity [14]. In addition, we also found that wetland ecosystems had the highest ESVs, which increased continuously during 1980–2010 (Table 6 and Table 7). Zhang et al. [45] found similar results in their study of Wuhan, and the main reason for this was that water bodies have the highest value coefficient and larger area, which could provide greater ecological benefits. However, the ESVs of 11 ecosystem services in farmland ecosystems and forestland ecosystems decreased with the reduction of natural vegetation such as farmland and forestland, which was consistent with the results of Dai et al. [46]. This was mainly because farmland was a major contributor to soil formation and conservation and food production, whereas forests played an important role in biodiversity conservation, climate regulation, and gas regulation [31,47]. In addition, desert ecosystems only provided a small portion of ecosystem services and their ESV has been decreasing during the study period, mainly due to the large reduction in bare land [21]. Overall, landscape changes in Wuhan have caused a rapid loss of ESVs, which is mainly due to the loss of ecological land. The conservation of ecological land and resources should be emphasized in the development of future landscape schemes.

### 5.3. Urban Expansion and Construction Suggestions

Urbanization is one of the main factors driving land use changes, which affects ecosystem services and raises serious environmental issues [13]. Wuhan, the largest city in central China and one of the 17 cities in the global sustainable development city plan issued by the United Nations Development Programme (UNDP) and the United Nations Environment Programme (UNEP), has experienced the dramatic expansion of urban built-up land [4]. This resulted in significant changes in Wuhan’s economic development, but also reduced the quality of ecosystem services and consumed large amounts of ecological land, including lakes, forestland, farmland, and grassland [17,20]. Therefore, we have proposed some constructive suggestions based on our findings to weigh the relationship between urbanization and ecological sustainability.

First, the results showed that the built-up land was mainly concentrated on the main urban area, and its continuous expansion intensified the loss of ecological land with a higher ESV. Therefore, we have proposed to establish green infrastructure in the main urban areas. Taking ecological protection as the primary goal and strengthening urban greening would improving the functions of ecosystem services such as climate regulation, environmental purification, and aesthetic landscapes. Secondly, there has also ben an expansion of urban construction within suburban areas in recent years, which has led to a loss of associated farmland. Thus, it is necessary to rationalize the use of urban land to alleviate the loss of farmland and improve the efficiency of the landscape. Specific measures include controlling the city’s scale, improving the economic efficiency of built-up land, and optimizing the land layout [17]. It would be beneficial to reclaim the unused land and grassland, converting it into irrigated farmland with high water accessibility, thereby increasing supply services for food production, as well as regulating services for carbon storage and nutrient retention [13]. Finally, the direction and speed of urbanization should be reasonably controlled to avoid blind urban expansion [48]. It is important to avoid ecologically destructive activities such as industrial land and built-up land in areas directly adjacent to natural ecosystems, thus reducing the encroachment on high-quality farmland and green space [21,45]. Eco-friendly landscapes such as parks and artificial green spaces should be increased.

### 5.4. Lanscape Planning of Ecological Emlemnts

**Figure 6:**
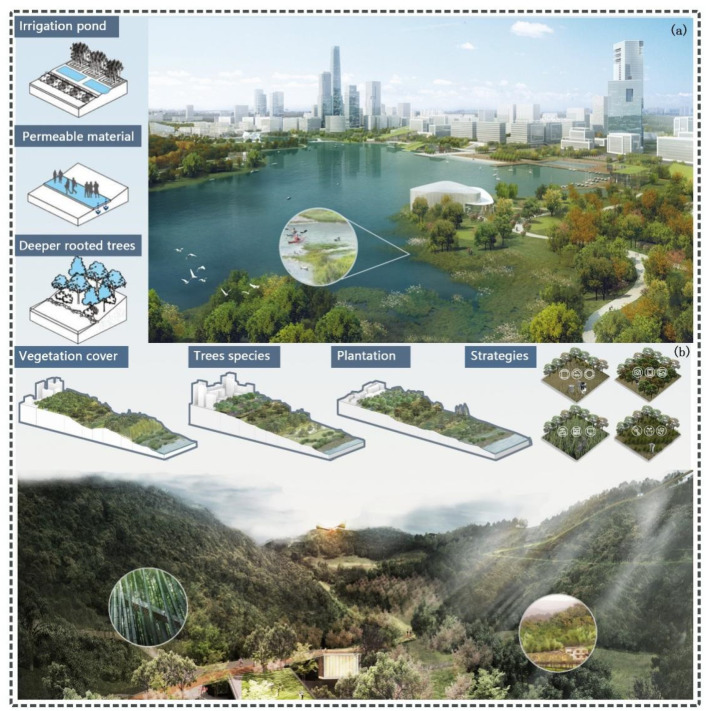
Landscape planning for waterscapes (**a**) and forestland (**b**) in Wuhan city, China.

In recent years, human activities such as urban expansion have increased the demand for water resources, while also leading to ecological problems such as water shortages and water quality deterioration [49]. The area of water bodies in Wuhan was gradually decreasing during 2010–2020. However, water bodies have a higher ESV, not only providing recreation for urban residents, but also helping to regulate regional climate and protect biodiversity [10]. As a result, this has led to a significant loss of ecosystem services and increased competition between ecological conservation and urban expansion [21]. To mitigate the loss of ESVs in the region, we have made some suggestions for waterscapes (Figure 6a). First, we suggested regulating the water environment according to the urban development conditions in Wuhan city. The implementation of water restoration projects should be strengthened in the main urban areas where built-up land is more concentrated. It is a priority to protect ecological spaces such as water and grassland, and to comprehensively address environment problems such as non-point-source pollution of lakes. In suburban areas with a high distribution of farmland and industrial land, it is advisable to strengthen the construction of sewage treatment facilities, so as to protect and improve water quality. This not only contributes to improved ecosystem health, but can also provide benefits to social development, providing services such as recreation, navigation, and flood control [50]. Second, the development of ecological landscapes, greenery protection, and urban construction should be considered comprehensively. Taking the protection of water resources as the core and drawing an ecological boundary line to create a high standard of urban ecological environment should also be considered. It could also be possible to reduce pollutants produced by industry, agriculture, and urban life through optimizing industrial structure, strengthening centralized wastewater treatment in industrial parks, and upgrading wastewater treatment plants [51]. Third, it is important to readjust the development of agriculture and aquaculture around the lake and to improve the connectivity of water bodies by using artificial dredging and other efforts, thus promoting water exchange and improving the ecological environment [52].

Meanwhile, forests play an important and unique role in enhancing ESV and habitat quality [39]. For forest landscapes, we suggest improving the level of regional afforestation and ecological protection by strengthening forestry management (Figure 6b). Firstly, it is necessary to increase forest coverage and optimize the selection of tree species. Secondly, this should be appropriately extended for the rotation period to make full use of the carbon sequestration potential of forests. Afforestation and reforestation to promote forest regeneration have also become ways to increase soil carbon sinks under human control. Finally, we propose reducing the increase in carbon sources by strictly controlling the occupation of construction land. At the same time, we should carefully promote the development of forest reserve resources and reduce the damage to forest land.

## 6. Conclusions

As a basis for resources, environmental, and ecological research and scientific management, the analysis of landscape change records the spatial and temporal dynamic characteristics of the land surface and the landscape altered by human activities. Landscape change can also affect the goods and services provided by ecosystems through changing the type and intensity of ecosystems. Therefore, an in-depth study of this topic can help researchers to understand the positive or negative impacts of various human activities on the ecological environment, and further reveal the relationship between changes in ecosystem services and land use changes. In this study, we used remote sensing to process Landsat TM/ETM+/OLI imageries, combined with kernel density analysis, to analyze the spatial and temporal characteristics of land use changes in Wuhan during 1980–2020. We also estimated the ESV of the region using an improved unit area value equivalent method to reveal the trends of ESV changes in Wuhan. The results showed that the land use in Wuhan had obvious changes during 1980–2020, mainly in terms of the decrease of farmland, forestland, and bare land, and the increase of built-up land and water bodies. Among them these the increase of built-up land and water bodies mainly came from farmland, bare land, and grassland, whereas the decrease of farmland was compensated for by ecological land such as grassland and forestland. In addition, the kernel density analysis indicated that the built-up land was mainly concentrated in the main urban areas, whereas the farmland was mainly distributed in the suburban areas. Water landscapes such as rivers and lakes were interspersed in the periphery of the main urban areas, but there was a significant decrease in their area during 2010–2020. Overall, there was a clear trend toward urban expansion in Wuhan, which also resulted in a significant loss of key ecosystem services. Therefore, we suggest that policy makers should focus on the balance between economic development and ecological protection when formulating landscape planning, and focus on controlling the expansion of built-up land as well as the reduction of ecological land. We also made some recommendations for urban expansion and landscape improvements in Wuhan based on the relationship between ecosystem service changes and landscape changes, thus providing reference information for land resource planning and ecological environment monitoring.

## Figures and Tables

**Table 1:** Categories and definitions of land use types in Wuhan city, China.

Land Use Type	Definition/Description
Farmland	Paddy field, glebe field, and other agricultural lands.
Forestland	Coniferous forest, broad-leaved forest, shrub, and mixed forest.
Grassland	Grass and meadows.
Water	Rivers, lakes, and pools.
Built-up land	Lands used for residential, industrial, commercial, transportation, etc.
Bare land	Bare rocks, riparian zones, and lands that are unused or difficult for using.

**Table 2:** Ecosystem service equivalent value per unit area (USD/ha).

Ecosystem Type		Farmland	Forest		Wetland	Desert	Urban
Land Use Type		Cultivated Land	Forestland	Grassland	Water	Bare Land	Built-Up Land
Provisioning services	Food production	661.83	141.13	184.16	389.31	0.00	0.00
	Raw material production	43.80	321.18	271.40	111.93	0.00	0.00
	Water supply	−1279.87	165.46	150.24	4034.27	0.00	0.00
Regulating services	Gas regulation	540.17	1056.02	954.74	374.72	9.73	0.00
	Climate regulation	277.39	3163.18	2524.97	1114.41	0.00	0.00
	Purify environment	82.73	939.22	833.58	2700.87	48.66	0.00
	Hydrological regulation	1323.67	2306.69	1851.32	49,754.37	14.60	0.00
Supporting services	Soil retention	4.87	1289.60	1163.14	452.58	9.73	0.00
	Nutrient cycling	92.46	97.33	87.24	34.07	0.00	0.00
	Biodiversity conservation	102.20	1172.81	1056.52	1240.94	9.73	0.00
Cultural services	Aesthetic landscape	43.80	515.84	465.25	919.76	4.87	0.00

**Table 3:** Area of six land use types in Wuhan city, China.

Land Use Type	1980		1990		2000		2010		2020	
	km^2^	%	km^2^	%	km^2^	%	km^2^	%	km^2^	%
Farmland	5714.93	66.65	5381.19	62.76	5235.26	61.05	4755.42	55.46	4526.66	52.79
Forestland	817.32	9.53	814.51	9.50	795.48	9.28	778.27	9.08	773.85	9.02
Grassland	80.19	0.94	72.41	0.84	72.22	0.84	78.49	0.92	79.53	0.93
Water	1320.11	15.40	1671.45	19.49	1737.56	20.26	1845.49	21.52	1814.17	21.16
Built-up land	495.83	5.78	550.49	6.42	658.49	7.68	1053.09	12.28	1319.82	15.39
Bare land	146.48	1.71	84.74	0.99	75.87	0.88	64.23	0.75	60.95	0.71

**Table 4:** The annual area of change (km^2^) and the net total area of change (km^2^) from 1980 to 2020 for six land use types.

Land Use Type	1980–1990	1990–2000	2000–2010	2010–2020	1980–2020
Farmland	−333.75	−145.93	−479.84	−228.75	−1188.27
Forestland	−2.81	−19.03	−17.21	−4.42	−43.47
Grassland	−7.77	−0.20	6.27	1.04	−0.66
Water	351.35	66.10	107.94	−31.32	494.06
Built-up land	54.66	107.99	394.60	266.73	823.99
Bare land	−61.74	−8.87	−11.64	−3.28	−85.53

**Table 5:** The annual rate of change (%) for six land use types and the dynamic degree (%) for the whole study area.

Indicators of Land Use Change		1980–1990	1990–2000	2000–2010	2010–2020	1980–2020
Annual rate of change	Farmland	−0.58	−0.27	−0.92	−0.48	−0.52
	Forestland	−0.03	−0.23	−0.22	−0.06	−0.13
	Grassland	−0.97	−0.03	0.87	0.13	−0.02
	Water	2.66	0.40	0.62	−0.17	0.94
	Built-up land	1.10	1.96	5.99	2.53	4.15
	Bare land	−4.21	−1.05	−1.53	−0.51	−1.46
Dynamic degree		0.95	0.41	1.19	0.62	3.07

**Table 6:** Ecosystem service total value of the study area in 1980, 1990, 2000, 2010, and 2020.

Ecosystem Service Type	1980		1990		2000		2010		2020	
	USD	%	USD	%	USD	%	USD	%	USD	%
Farmland ecosystem	1.08 × 10^9^	10.67	1.02 × 10^9^	8.34	9.91 × 10^8^	7.88	9.00 × 10^8^	6.86	8.57 × 10^8^	6.65
Forestland ecosystem	9.89 × 10^8^	9.75	9.79 × 10^8^	8.01	9.57 × 10^8^	7.62	9.44 × 10^8^	7.19	9.40 × 10^8^	7.30
Wetlands ecosystem	8.07 × 10^9^	79.56	1.02 × 10^10^	83.64	1.06 × 10^10^	84.49	1.13 × 10^10^	85.94	1.11 × 10^10^	86.05
Desert ecosystem	1.43 × 10^6^	0.01	8.25 × 10^5^	0.01	7.38 × 10^5^	0.01	6.25 × 10^5^	0	5.93 × 10^5^	0
Urban ecosystem	0	0	0	0	0	0	0	0	0	0
Total value	1.01 × 10^10^	100	1.22 × 10^10^	100	1.26 × 10^10^	100	1.31 × 10^10^	100	1.29 × 10^10^	100

**Table 7:** Ecosystem service value ($) changes of different ecosystem services.

Year	Ecosystem Service Types	Ecosystem Types					Sum
		Farmland Ecosystem	Forest Ecosystem	Wetland Ecosystem	Desert Ecosystem	Urban Ecosystem	
1980–1990	FP	−2.21 × 10^7^	−1.83 × 10^5^	1.37 × 10^7^	0.00	0.00	−8.59 × 10^6^
	RMP	−1.46 × 10^6^	−3.01 × 10^5^	3.93 × 10^6^	0.00	0.00	2.17 × 10^6^
	WS	4.27 × 10^7^	−1.63 × 10^5^	1.42 × 10^8^	0.00	0.00	1.84 × 10^8^
	GR	−1.80 × 10^7^	−1.04 × 10^6^	1.32 × 10^7^	−6.01 × 10^4^	0.00	−5.96 × 10^6^
	CR	−9.26 × 10^6^	−2.85 × 10^6^	3.92 × 10^7^	0.00	0.00	2.70 × 10^7^
	PE	−2.76 × 10^6^	−9.12 × 10^5^	9.49 × 10^7^	−3.00 × 10^5^	0.00	9.09 × 10^7^
	HR	−4.42 × 10^7^	−2.09 × 10^6^	1.75 × 10^9^	−9.01 × 10^4^	0.00	1.70 × 10^9^
	SR	−1.63 × 10^5^	−1.27 × 10^6^	1.59 × 10^7^	−6.01 × 10^4^	0.00	1.44 × 10^7^
	NC	−3.09 × 10^6^	−9.52 × 10^4^	1.20 × 10^6^	0.00	0.00	−1.98 × 10^6^
	BC	−3.41 × 10^6^	−1.15 × 10^6^	4.36 × 10^7^	−6.01 × 10^4^	0.00	3.90 × 10^7^
	AL	−1.46 × 10^6^	−5.07 × 10^5^	3.23 × 10^7^	−3.01 × 10^4^	0.00	3.03 × 10^7^
1990–2000	FP	−9.66 × 10^6^	−2.72 × 10^5^	2.57 × 10^6^	0.00	0.00	−7.36 × 10^6^
	RMP	−6.39 × 10^5^	−6.16 × 10^5^	7.40 × 10^5^	0.00	0.00	−5.16 × 10^5^
	WS	1.87 × 10^7^	−3.18 × 10^5^	2.67 × 10^7^	0.00	0.00	4.50 × 10^7^
	GR	−7.88 × 10^6^	−2.03 × 10^6^	2.48 × 10^6^	−8.63 × 10^3^	0.00	−7.44 × 10^6^
	CR	−4.05 × 10^6^	−6.07 × 10^6^	7.37 × 10^6^	0.00	0.00	−2.75 × 10^6^
	PE	−1.21 × 10^6^	−1.80 × 10^6^	1.79 × 10^7^	−4.32 × 10^4^	0.00	1.48 × 10^7^
	HR	−1.93 × 10^7^	−4.42 × 10^6^	3.29 × 10^8^	−1.30 × 10^4^	0.00	3.05 × 10^8^
	SR	−7.11 × 10^4^	−2.48 × 10^6^	2.99 × 10^6^	−8.63 × 10^3^	0.00	4.36 × 10^5^
	NC	−1.35 × 10^6^	−1.87 × 10^5^	2.25 × 10^5^	0.00	0.00	−1.31 × 10^6^
	BC	−1.49 × 10^6^	−2.25 × 10^6^	8.20 × 10^6^	−8.63 × 10^3^	0.00	4.45 × 10^6^
	AL	−6.39 × 10^5^	−9.90 × 10^5^	6.08 × 10^6^	−4.32 × 10^3^	0.00	4.45 × 10^6^
2000–2010	FP	−3.18 × 10^7^	−1.27 × 10^5^	4.20 × 10^6^	0.00	0.00	−2.77 × 10^7^
	RMP	−2.10 × 10^6^	−3.83 × 10^5^	1.21 × 10^6^	0.00	0.00	−1.28 × 10^6^
	WS	6.14 × 10^7^	−1.91 × 10^5^	4.35 × 10^7^	0.00	0.00	1.05 × 10^8^
	GR	−2.59 × 10^7^	−1.22 × 10^6^	4.04 × 10^6^	−1.13 × 10^4^	0.00	−2.31 × 10^7^
	CR	−1.33 × 10^7^	−3.86 × 10^6^	1.20 × 10^7^	0.00	0.00	−5.14 × 10^6^
	PE	−3.97 × 10^6^	−1.09 × 10^6^	2.92 × 10^7^	−5.66 × 10^4^	0.00	2.40 × 10^7^
	HR	−6.35 × 10^7^	−2.81 × 10^6^	5.37 × 10^8^	−1.70 × 10^4^	0.00	4.71 × 10^8^
	SR	−2.34 × 10^5^	−1.49 × 10^6^	4.88 × 10^6^	−1.13 × 10^4^	0.00	3.15 × 10^6^
	NC	−4.44 × 10^6^	−1.13 × 10^5^	3.68 × 10^5^	0.00	0.00	−4.18 × 10^6^
	BC	−4.90 × 10^6^	−1.36 × 10^6^	1.34 × 10^7^	−1.13 × 10^4^	0.00	7.12 × 10^6^
	AL	−2.10 × 10^6^	−5.96 × 10^5^	9.93 × 10^6^	−5.67 × 10^3^	0.00	7.22 × 10^6^
2010–2020	FP	−1.51 × 10^7^	−4.32 × 10^4^	−1.22 × 10^6^	0.00	0.00	−1.64 × 10^7^
	RMP	−1.00 × 10^6^	−1.14 × 10^5^	−3.51 × 10^5^	0.00	0.00	−1.47 × 10^6^
	WS	2.93 × 10^7^	−5.75 × 10^4^	−1.26 × 10^7^	0.00	0.00	1.66 × 10^7^
	GR	−1.24 × 10^7^	−3.67 × 10^5^	−1.17 × 10^6^	−3.19 × 10^3^	0.00	−1.39 × 10^7^
	CR	−6.35 × 10^6^	−1.14 × 10^6^	−3.49 × 10^6^	0.00	0.00	−1.10 × 10^7^
	PE	−1.89 × 10^6^	−3.28 × 10^5^	−8.46 × 10^6^	−1.60 × 10^4^	0.00	−1.07 × 10^7^
	HR	−3.03 × 10^7^	−8.27 × 10^5^	−1.56 × 10^8^	−4.79 × 10^3^	0.00	−1.87 × 10^8^
	SR	−1.11 × 10^5^	−4.49 × 10^5^	−1.42 × 10^6^	−3.19 × 10^3^	0.00	−1.98 × 10^6^
	NC	−2.12 × 10^6^	−3.39 × 10^4^	−1.07 × 10^5^	0.00	0.00	−2.26 × 10^6^
	BC	−2.34 × 10^6^	−4.09 × 10^5^	−3.89 × 10^6^	−3.19 × 10^3^	0.00	−6.64 × 10^6^
	AL	−1.00 × 10^6^	−1.80 × 10^5^	−2.88 × 10^6^	−1.60 × 10^3^	0.00	−4.06 × 10^6^

Note: FP, food production; RMP, raw material production; WS, water supply; GR, gas regulation; CR, climate regulation; PE, environmental purification; HR, hydrological regulation; SR, soil retention; NC, nutrient cycling; BC, biodiversity conservation, AL, aesthetic landscape.
